# Assessing a Digital Public Health Intervention: Survey Implementation and Analysis in Washington State Using an Address-Based Sample

**DOI:** 10.1055/a-2695-8451

**Published:** 2025-09-23

**Authors:** Adam S. Elder, Andrea R. Molino, Rebecca A. Hills, Debra Revere, Chris Baumgartner, Bryant T. Karras, Janet G. Baseman

**Affiliations:** 1 Department of Epidemiology, School of Public Health, University of Washington, Seattle, Washington, United States; 2 Department of Health Systems and Population Health, School of Public Health, University of Washington, Seattle, Washington, United States; 3 Department of Health, Washington State, Tumwater, United States

**Keywords:** smart phone, Electronic health records and systems, information assurance, public health, statistical methods, General information systems and technologies in clinical settings

## Abstract

**Background:**

In November 2021, the Washington State Department of Health launched WA Verify, a quick-response (QR) code-based tool that allows users to share their coronavirus disease 2019 (COVID-19) vaccine status using a mobile phone.

**Objectives:**

This study aimed to gather an unbiased sample of Washington residents to collect information about their experiences and opinions on digital public health interventions, with a particular focus on WA Verify.

**Materials and Methods:**

The WA Verify evaluation team designed and implemented a statewide survey using an address-based sample to assess respondents' knowledge, attitudes, and practices regarding COVID-19, WA Verify, and technology. To facilitate comparisons with known characteristics of the Washington State population, the survey included questions also found in state and national surveys and the U.S. Census. Weights based on demographic characteristics were developed to allow for weighted analyses in the future.

**Results:**

The unweighted demographic distribution of respondents mostly matched that of the Washington State population, with the largest discrepancy in education; 58.9% of respondents reported having a four-year degree or higher versus 37.3% of the state population. Across race, education level, age and gender, there were differences in reported experiences with and views on requirements to provide vaccination verification and awareness and use of WA Verify.

**Conclusion:**

Despite differences in experiences and views on WA Verify, there is broad interest in the tool, suggesting opportunities to expand its adoption. Data and information gathered with this survey, conducted using an address-based sample, provide a strong foundation for further analyses to inform public health interventions, particularly informatics-focused projects.

## Background and Significance

During the height of the coronavirus disease 2019 (COVID-19) pandemic, public health mandates limited social contact and travel, compelling the implementation of measures to monitor and manage behavior that could impact public health. 1 2 As vaccines and boosters became available, restrictions were gradually relaxed; in some cases, proof of vaccination or a negative COVID-19 test result was required to participate in activities or enter venues or businesses.

Providing proof of vaccination status took many forms. Initially, most individuals provided the paper CDC vaccine card as proof of vaccination. 3 Subsequently, after a rapid development process, Washington (WA) state along with other U.S. states and several countries provided access to mobile tools developed to facilitate sharing of COVID-19 vaccine status. Washington's Verifiable Clinical Information (VCI) tool is called WA Verify and was built on the SMART Health Card framework, 4 5 6 providing a convenient means for individuals to obtain and show proof of their COVID-19 vaccine status. To use WA Verify residents may visit the tool's Web site and enter their name, date of birth, and either an email or phone number. Users then receive a QR code via the entered contact information. This code can be stored on a smartphone or printed on a piece of paper.

Prior to roll-out, little empirical evidence had been collected on expected acceptance and uptake of a tool like WA Verify. Although there are several examples of vaccine verification tools implemented across the country, 7 8 9 reports on uptake, acceptance and usage practices are scant. The acceptability of a hypothetical tool to show vaccination status was considered in multiple studies and these studies found that interest in the tool ranged from 60–80%, though acceptability of requirements for sharing vaccination status imposed by various entities was far lower. 10 11 12 13 14 Some have also considered ethical and economic concerns related to policies requiring proof of vaccination for entrance to dining or entertainment venues. 15 16 17 However, WA Verify was designed as an alternative method of providing vaccination information rather than a policy or stance regarding whether or not proof of vaccination should be required.

Soon after the November 2021 system launch, WA Verify saw large numbers of requests from WA residents seeking access to their vaccine records through the tool. Although millions of records requests have now been made, due to its rapid implementation, little was known about how the public received the tool, barriers to acceptance and utilization, or the factors that influenced adoption. More broadly, little was known about how WA state residents interact with and feel about technology related to public health in general. To address this knowledge gap and to evaluate acceptance of WA Verify, a survey targeting WA residents was developed and administered.

This survey and its results represent one of the first comprehensive reports on the knowledge, attitudes, and practices surrounding a digital vaccine verification technology that has been widely implemented at the state level. This large, methodologically robust survey provides a detailed description of WA state and can inform implementation of useful tools that are accepted by the community, promote health equity and improve health outcomes. While many high-level results are presented here, further analyses have been conducted and can be found both in the appendix and in other published work. 18

## Objectives

This study sought to inform the development of WA Verify through the implementation and analysis of a representative, address-based survey of Washington residents. Specifically, the survey and corresponding analysis focused on tech literacy, experiences with vaccine verification and knowledge and opinions regarding WA Verify and public health policies meant to limit the spread of COVID-19.

## Materials and Methods

The survey was designed and analyzed by the University of Washington's WA Verify evaluation team in the School of Public Health in partnership with the WA State Department of Health (WA DOH). The Social and Economic Sciences Research Center (SESRC) a at Washington State University implemented the survey, carrying out sampling as well as printing, translation, web hosting, mail distribution, and data entry.

### Survey Design

A brief survey was designed for both paper/mail and electronic completion. Survey questions were developed iteratively and focused on the following areas: experiences with COVID-19, situations requiring proof of vaccination or testing, barriers and facilitators to use of public health digital tools, and basic demographic information. After an iterative design and review process, the survey included 32 questions and took approximately 10 minutes to complete. The complete survey instrument can be found in the Supplemental Files .

### Data Collection

**Fig. 1:**
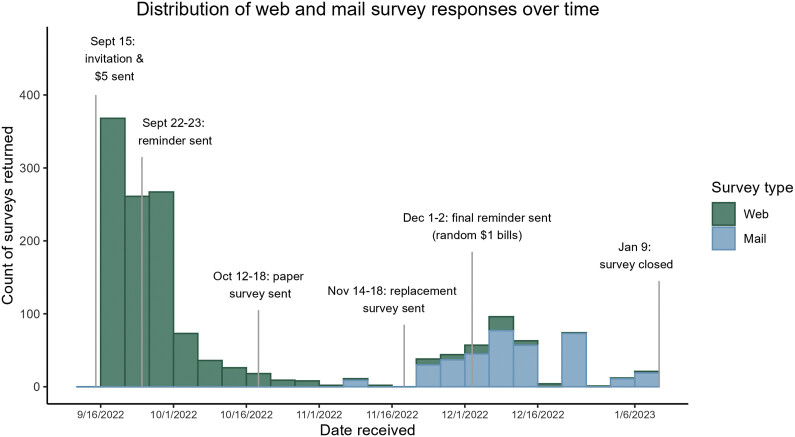
Distribution of Web and mail survey responses over time. On September 15, 2022, postal invitation letters were mailed to all 5,000 addresses in the study sample. Each bar represents 5 days. The first survey was received on September 16, 2022 and the last included survey was received on January 9, 2023.

The target population was adult residents of WA state. A simple random sample of 5,000 addresses was obtained from a database based on the United States Postal Services Delivery Sequence File 19 with a 97% coverage rate. On September 15, 2022, letters were mailed to all 5,000 addresses in the study sample. The initial invitation letter informed recipients about the survey and included instructions for completing the survey online. The initial mailing ( Supplementary Material ) included links to the online instrument and a $5 bill as an incentive. One week after the initial mailing, a reminder postcard was sent to those who had not yet completed the survey). On October 12 and November 14, a paper questionnaire, cover letter, and postage-paid return envelope were mailed to all addresses for which the survey had not yet been completed online. A Spanish language translation of the letter was also included in the November 14 mailing. A final reminder was sent to nonrespondents on December 1, 2022, with a random subset receiving $1 bills with the mailing. The survey closed on January 9, 2023. More details about the distribution timeline are described in the Appendix and shown in Fig. 1 .

### Statistical Analysis

The demographic characteristics of the survey sample were summarized using sample proportions. Additionally, these sample proportions were compared with those of WA state as described by the United States Census Bureau, American Community Survey 2021 5-year estimates. 20 21 22 23 24 25 26 27 In an effort to improve further analyses, calibration weights were also created for the dataset based on race, ethnicity, age, and sex. Based on these weights, we estimated the number of WA Verify users in Washington and compared this estimate to the number of QR code download requests, which is known exactly. For more details on our weighting method ( Supplementary material ).

To better understand the barriers and facilitators to use of digital verification in the state, comparisons of four outcomes were considered across multiple demographic factors. The outcomes included: experience being asked for proof of vaccination or a negative test, hearing about WA Verify, using WA Verify, and support of vaccination or testing requirements. The demographic characteristics considered were age, gender, region of Washington (East vs. West), race and ethnicity, and education level. The two regions of Washington considered here are based on county lines with the division largely dictated by the Cascade Mountain range passing through the state ( Supplementary Fig. S7 ). The eastern portion of the state is less metropolitan 28 with a different economic makeup. As a result of this and other factors, the eastern region of Washington is generally more conservative, less well educated, has higher rates of poverty, and lower rates of employment than the western region ( Table 1 ). Eastern versus Western Washington regional comparisons are often informative for local public health practice to better tailor interventions to the population served.

**Table 1:** Demographic differences between Western and Eastern Washington

Covariate, ACS table code a	Region	
	West	East
Percentage of Individuals over 25 with a bachelor's degree or more, B15003	40	27
Percentage of families with an income below the poverty line in the past year in Washington, B17010	6	9
Percent of individuals over 25 who are in the labor force, B16010	66	61
Percent of all individuals living in a rural environment b	87	72

a All statewide measures are taken from the American Community Survey Data. Data from the 5-year estimates for 2021 are used throughout with the code for the specific table used given in the first column.

b Based on the U.S. Census Bureau's urban–rural classification

R version 4.3.2 (2023-10-31) and the R package Dplyr 1.1.4 was used. Descriptive data were presented as counts and percentages.

## Results

Of the 5,000 invitations mailed, 1,491 households responded to the survey, and 302 invitations were returned as undeliverable. The response rate for the survey (defined as the number of fully and partially completed surveys divided by the number surveys not returned) 29 was 32%. Three-quarters of the respondents (1,133, 76%) completed the survey online; 358 (24%) completed and returned the paper survey. All respondents completed the English language version of the survey; none of the Spanish translated questionnaires were returned.

### Demographics

For many of the demographic characteristics, including race/ethnicity, disability status, household size, and WA region (East vs. West), the survey sample proportions were comparable to WA state as a whole. There were, however, some exceptions. The sample had a lower proportion of male respondents (37.8%) than WA state (50.3%). Relative to WA state, the sample included fewer young individuals 18 to 29 years (sample: 9.0% vs. WA: 20.7%) and had a higher proportion of older individuals 70 to 79 years (sample:15.7% vs. WA: 8.7%). In the case of educational attainment, the sample had a higher proportion (56.2%) of “4-year degree or more” graduates compared with the state overall (37.3%). A smaller proportion of our survey respondents spoke a language other than English at home (11.3%) compared with WA state as a whole (20.3%).

Table 2 presents the weighted and unweighted sample proportions as well as the reference proportion for the state average for each of the considered variables. The last column of the table lists the ratio of the sample proportion (both weighted and unweighted) to the reference; values closer to 1 indicate a more representative sample. It is noteworthy that while some variables (such as language spoken at home) better match the reference when weighted, others see little improvement from weighting (e.g., education).

**Table 2:** Demographics of the unweighted and weighted samples compared with WA state demographics

Attribute, ACS table code	Values/range	Count	Percent a	Weighted percentage	Washington statewide percentages b	Survey percent over state percent: unweighted (weighted)
Gender, B01001	Female	813	58.7	50.0	50.0 c	1.19 (1.00)
	Male	563	40.7	50.0	50.0 c	0.81 (1.00)
	Transgender	3	0.2			
	Nonbinary/nonconforming	5	0.4			
	Prefer not to respond	44				
	Missing	63				
Age, B01001	18–29	134	9.4	20.7	20.7	0.44 (1.00)
	30–39	221	15.5	19.0	19.0	0.83 (1.00)
	40–49	205	14.4	16.3	16.3	0.89 (1.00)
	50–59	234	16.4	16.0	16.0	1.02 (1.00)
	60–69	315	22.1	15.0	15.0	1.48 (1.00)
	70–79	234	16.4	8.7	8.7	1.93 (1.00)
	80+	84	5.9	4.3	4.3	1.32 (1.00)
	Missing	64				
Race and ethnicity, B03002	American Indian or Alaska Native (AIAN) alone	4	0.3	0.4	0.9	0.32 (0.43)
	Asian alone	112	8.2	10.2	8.9	0.92 (1.15)
	Black alone	36	2.6	3.4	3.7	0.71 (0.92)
	Hispanic/Latinx any race	75	5.5	13.2	13.2	0.41 (1.00)
	Native Hawaiian and Other Pacific Islander (NHOPI) alone	6	0.4	0.5	0.6	0.68 (0.74)
	Two or more races specified	60	4.4	5.0	5.8	0.76 (0.86)
	Some other race alone	17	1.2	0.8	0.4	3.23 (2.21)
	White alone	1,060	77.4	66.5	66.5	1.16 (1.00)
	Missing	121				
Highest level of education, B15003	Less than high school	13	0.9	1.4	8.1	0.11 (0.17)
	High school graduate	187	13.2	13.2	21.8	0.52 (0.55)
	2-y degree or some college	384	27.0	26.7	32.8	0.82 (0.81)
	4-y degree or more	838	58.9	58.7	37.3	1.58 (1.57)
	Missing	69				
Disability, B18101	Yes	197	14.7	12.6	13.7	1.08 (0.92)
	No	1140	85.3	87.4	86.3	0.99 (1.01)
	Prefer not to respond	89				
	Missing	65				
Speak language other than English at home, B16001 21	Yes	168	11.9	19.7	20.3	0.59 (0.97)
	No	1240	88.1	80.3	79.7	1.10 (1.01)
	Missing	83				
Household size, B08201	One (live alone)	324	22.8	10.1	10.5 d	2.18 (0.97)
	Two	608	42.8	33.0	27.8 d	1.54 (1.19)
	Three	213	15.0	18.3	18.2 d	0.82 (1.00)
	Four or more	275	19.4	38.6	43.5 d	0.44 (0.89)
	Missing	71				
Parent or guardian to child under 18, B11003	Yes	336	23.6	34.0	42.7	0.55 (0.80)
	No	1086	76.3	66.0	57.3	1.33 (1.15)
	Missing	69				
Region of Washington, B01001	Eastern	283	19.0	18.3	21.7	0.88 (0.84)
	Western	1208	81.0	81.7	78.3	1.03 (1.04)

a Calculated percentages do not include missing values.

b All statewide measures are taken from the American Community Survey Data. Data from the 5-year estimates for 2021 are used throughout, with the code for the specific table used given in the first column.

c Note that census percentages are based on only those 18 and older to match the joint age–sex distribution used when creating weights.

d Household size is reported by the census as a per-household average. This average is adjusted to be a per-person average with 4 or more individual households being assumed to have an average of 4.9 individuals (selected to align with the population count).

While the exact number of individuals using WA Verify is not known, the WA DOH reported that QR codes had been downloaded 1.7 million times between October 25, 2021, and January 9, 2022. Although the number of downloads is expected to be larger than the number of WA Verify users because individuals can request QR codes multiple times, it is roughly in line with the estimated 26.4% of WA state residents (18 or older) using WA Verify from the weighted survey, which would correspond to roughly 1.5 million users in the state.

### Experiences with Coronavirus Disease 2019 Vaccine Verification

Roughly two-thirds of respondents (66.5%) reported being asked for proof of COVID-19 vaccination or a negative COVID-19 test during the 12 months prior to receiving the survey. A little over half of respondents (54.0%) had not heard of WA Verify before receiving the survey, and a majority (72.8%) reported they did not use WA Verify or a similar tool ( Table 3 ).

**Table 3:** Experience with vaccine verification, familiarity with, and use of WA Verify

Question	Yes (%)	No (%)	No, but use similar tool	Missing (%)
In the last 12 mo, have you been asked to show proof of COVID-19 vaccination, or a negative COVID-19 test result before participating in an activity or entering a business? (Q06)	991 (66.5%)	481 (32.3%)	–	19 (1.3%)
Before receiving this survey, had you heard about WA Verify? (Q08)	665 (44.6%)	805 (54.0%)	–	21 (1.4%)
Do you use WA Verify? (Q12)	309 (20.7%)	1085 (72.8%)	50 (3.4%)	47 (3.2%)

### Situations Requiring Proof of Vaccination and/or Negative Test Results

**Fig. 2:**
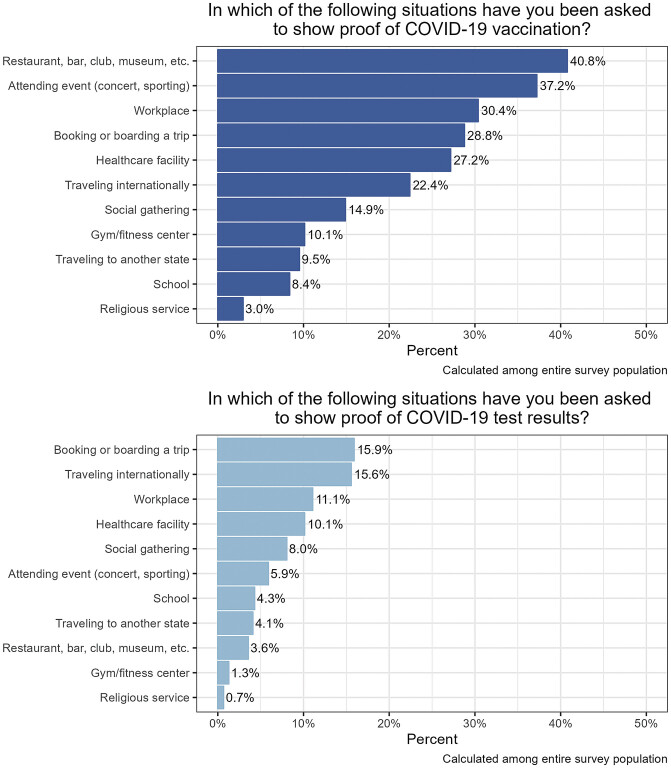
Situations where respondents were asked for proof of vaccination or negative test results.

Requests for proof of vaccination were primarily reported before entering establishments, attending events, entering the workplace, and when booking travel or prior to boarding. Far fewer respondents reported requests for negative COVID-19 tests, and such requests were most often made when booking travel, entering the workplace or visiting a health care facility. Fig. 2 shows more details on reported requests for proof of vaccination and negative test results.

### Barriers and Facilitators to Using Public Health Digital Tools

**Fig. 3:**
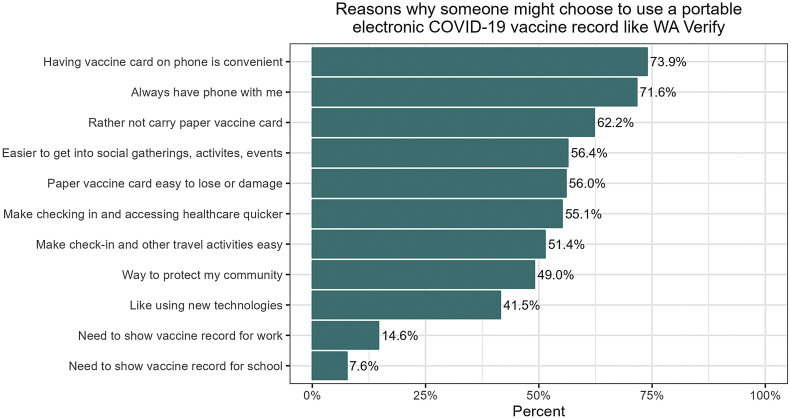
Reasons to use a tool like WA Verify. Reported percentages are for responses to “Reasons why someone might choose to use a portable electronic COVID-19 vaccine record like WA Verify.” While 3.6% of participants wrote in a response, they are not listed here.

**Fig. 4:**
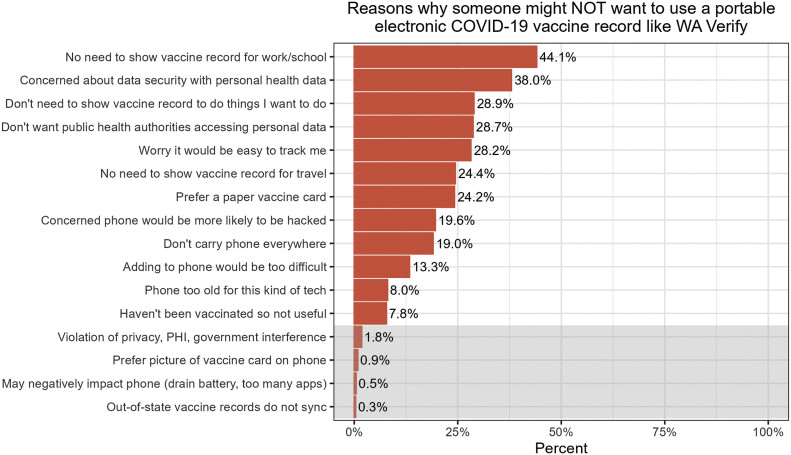
Reasons to not use a tool like WA Verify. Reported percentages are for responses to “Reasons why someone might NOT want to use a portable electronic COVID-19 vaccine record like WA Verify.” Options in gray were created based on participant write-in responses and 1.9% of participants wrote in a reason not listed above.

When asked about reasons to use a tool like WA Verify, the most common responses included the tool's convenience (73.9%) ease of access to events and venues (56.4%), making health care check-ins and access easier (55.1%) and as a good way to protect one's community (49.0%). Primary reasons for not using a tool like WA Verify included not needing to show vaccine status for school or work (44.1%) or other activities (28.9%), concerns about data security (38%) and not wanting public health authorities accessing personal data (28.7%). Figs. 3 and 4 summarize reasons for using and not using a tool like WA Verify, respectively.

**Fig. 5:**
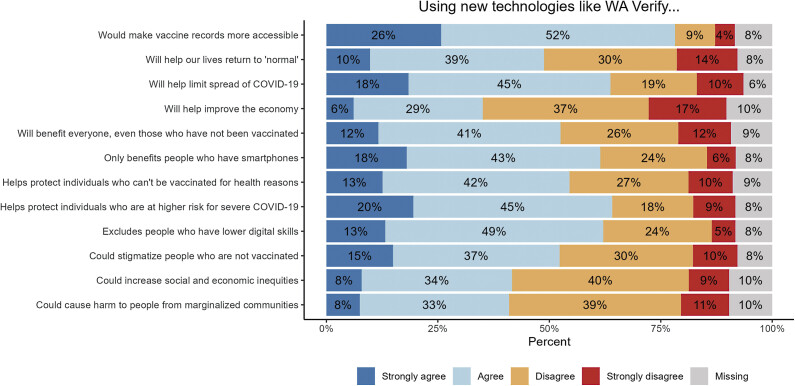
Opinions about public health technology like WA Verify. Reported percentages are for responses to “Using new technologies like WA Verify….”

Many respondents (74.6%) supported public health policies requiring proof of vaccination or negative COVID-19 test results. Although there was agreement that a technology like WA Verify would help limit the spread of COVID-19 (63%), there was also concern that only smartphone owners would benefit from such a tool (61%) and those with lower digital skills would be left out (62%). Opinions on other possible positives and negatives of digital health technology are summarized in Fig. 5 .

**Fig. 6:**
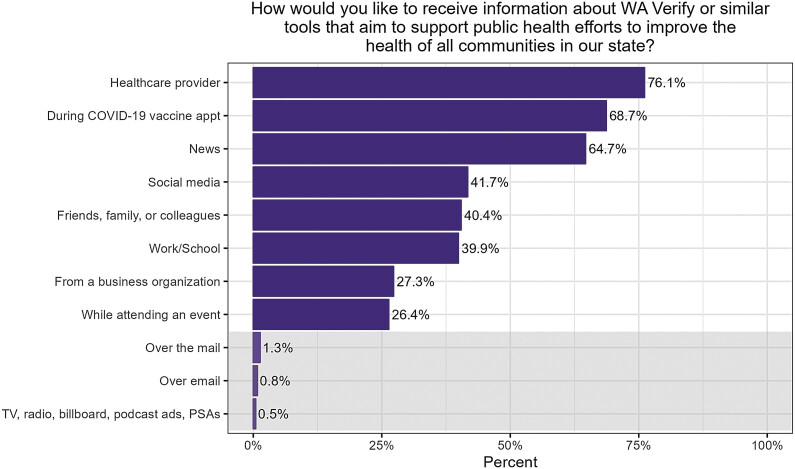
Communication preferences for receiving information about WA Verify or similar tools. Reported percentages are for responses to “How would you like to receive information about WA Verify or similar tools that aim to support public health efforts to improve the health of all communities in our state?” Options in gray were created based on participant write-in responses and 2% of participants wrote in a source to receive information not listed above.

Survey respondents were also asked how they would prefer to have information shared with them. Health care-related sources—either from health care providers (76.1%) or during COVID-19 vaccine appointments (68.7%)—were the most popular. More than half of respondents indicated they would like to receive public health-related information through the news (64.7%). The popularity of other communication sources is summarized in Fig. 6 .

Among the notable trends in awareness of, use of and interest in WA Verify, younger individuals and more highly educated individuals were more likely to have been asked for proof of vaccination, have heard of WA Verify, and to be using WA Verify than their older and less educated counterparts. Additionally, support for requiring proof of vaccination was higher among Asian individuals (94%) compared with other races. While differences across all groups were observed, substantive differences were seen between the two regions of Washington for each of the considered questions ( Table 4 ).

**Table 4:** Frequency of demographic characteristics and within-group experiences regarding vaccine and test verification, awareness, use, and support of WA Verify

	Group size	Percent asked for proof a	Percent heard WA Verify b	Percent use WA Verify c	Percent supporting WA Verify d
Age					
18–29	134	83	43	19	79
30–39	221	79	48	25	74
40–49	205	74	58	33	73
50–59	234	72	51	23	80
60–69	315	60	48	23	78
70–79	234	58	35	12	81
80+	84	48	27	9	83
Region e					
Eastern	283	46	37	15	63
Western	1,208	72	47	23	81
Education					
Less than high school	13	23	23	8	77
High school graduate or GED	187	46	25	10	71
2-y degree or some college	384	55	40	18	72
4-y degree or more	838	80	53	27	82
Race and ethnicity					
AIAN alone	4	25	0	0	50
Black alone	36	78	39	17	83
Asian alone	112	81	49	31	94
NHPI alone	6	67	50	17	67
Hispanic any race	75	69	44	16	77
Two or more races specified	60	64	53	23	63
Another race	17	71	41	6	35
White alone	1,060	67	46	22	79
Gender					
Female	813	68	49	23	81
Male	563	69	42	22	76
Transgender	3	67	0	0	100
Nonbinary/nonconforming	5	80	40	20	100
Prefer not to respond	44	55	27	0	24

a Survey Question: In the last 12 months, have you been asked to show proof of COVID-19 vaccination, or a negative COVID-19 test result before participating in an activity or entering a business?

b Survey Question: Before receiving this survey, had you heard about WA Verify?

c Survey Question: Do you use WA Verify?

d Survey Question: How do you feel about policies that require proof of vaccination or a negative COVID-19 test result to enter spaces that are high risk of COVID-19 spread?

e Region is determined by county ( Supplemental Fig. S7 ).

### Regionality of Verification Experiences and Barriers and Facilitators

Respondents living in Eastern and Western WA differed in their experiences surrounding vaccine verification and concerns with using a public health digital tool like WA Verify. A larger proportion of those from Western WA reported they had been asked for proof of COVID-19 vaccine or test in the past 12 months compared with those in Eastern WA (72.2 vs. 46.2%). Similarly, a greater proportion of those from Western WA reported having heard of WA Verify before the survey (47.1 vs. 37.2%) and had used WA Verify or a similar tool (22.9 vs. 15.0%).

Although over three-quarters of respondents (77%) expressed support (“strongly support” or “support”) for COVID-19 vaccination or testing requirements, the level of support was greater in Western WA (81%) compared with Eastern WA (63%).

Among respondents who did not report using WA Verify or a similar tool ( *N* = 1,085, 72.8%), 36% reported being willing and 27% reported being somewhat willing to use a portable COVID-19 vaccine record. Comparing state regions, a larger proportion of those in Western WA were “willing” or “somewhat willing” to use a portable COVID-19 vaccine record (66 vs. 53%, respectively).

## Discussion

The WA Verify statewide survey described here sought to provide an accurate snapshot of the WA state population, gathering data from a large and representative sample regarding a specific tool (WA Verify) and digital health tools in general. The survey found that a majority (66.5%) of respondents had been asked for proof of vaccination in the past 12 months. Additionally, close to half (44.6%) of respondents had heard of the WA Verify tool and 20.7% of respondents reported using the tool. While survey percentages cannot be directly translated into population percentages, these results suggest that this rapidly deployed tool was quickly adopted by millions of residents who saw the value in both the tool and the public health policies it supported.

In addition to awareness and adoption of WA Verify, other key findings may help guide further development of this or similar tools. First, WA Verify adoption varied by region with 22.9% of Western Washington residents (in the more metropolitan part of the state) versus 15.0% of Eastern Washington residents reporting use of the tool. Notably, this difference in adoption is roughly proportional to the difference between the regions reported needs to present proof of vaccines or a negative test (72.2% in the West vs. 46.2% in the East). Respondents indicated interest in the tool with 63% of respondents who didn't use WA Verify reporting at least some willingness to use the tool.

While others have provided system descriptions 7 8 9 and formative work on perceived acceptability of vaccine verification tools, 11 15 the study presented here is one of the few examples of an implemented immunization verification tool and corresponding comprehensive survey supporting its continued use and improvement.

The large, population-based survey presented here had many strengths but also had several limitations. Participation was voluntary and while efforts were made to present the survey and its questions in a neutral manner, response bias resulting from under or overrepresentation of certain groups could not be fully accounted for, even with our weighting strategy. As an example, despite providing a Spanish-language version of the survey, there were no completed versions of this survey returned, representing one of many possible forms of nonresponse bias. Opinions on vaccination and public health policies related to COVID-19 are often influenced by ideological perspectives and political affiliation. To maintain a neutral tone, the survey did not include questions in these potentially sensitive areas, limiting our ability to explore these differences. Additionally, this study was conducted during a period in which interest in and efforts to mitigate COVID-19 were greater than they are now, meaning the opinions captured may not reflect current views.

## Conclusion

Insights gained from surveys such as the one presented here are invaluable in tailoring messaging and system design, particularly if data are integrated early in the development process. As the public health landscape evolves and digital health solutions become more prevalent, leveraging the insights gained from surveys—such as the one presented here—will be an important tool for understanding the population of interest and guiding the practice of public health informatics. As examples, additional analysis and a set of personas (a user-centered design tool) were developed from the WA Verify survey data to help guide future public health messaging. 18 Ultimately, this targeted, data-focused strategy not only improves engagement but also ensures that technological advancements in public health are responsive to the actual needs and preferences of the communities they aim to serve.

## Clinical Relevance Statement

This study highlighted the value of simple random address-based samples for obtaining representative data on communities or geographic areas; this technique should be considered as a part of formative evaluation for community-based health informatics projects. Survey results indicate that the mobile vaccine verification tool deployed in WA state enjoys broad appeal and acceptance. Regional differences in both public health opinions and willingness to adopt were also noted and should be considered in informational and marketing campaigns.

## Footnotes

a https://sesrc.wsu.edu/
