# Supplementary material for: Assessing a Digital Public Health Intervention: Survey Implementation and Analysis in Washington State Using an Address-Based Sample
Source: ACI open. 2025 Sep 23;9(2):e54–64. doi: 10.1055/a-2695-8451 (PMC13393551; doi:10.1055/a-2695-8451)
Supplement: Supplementary file 1 — Supplementary Material [file 10-1055-a-2695-8451_27074473.pdf]

Supplementary Material

Additional Details of Survey Distribution and Data Collection

The target population was adult residents of WA state. A simple random sample of 5,000 residential household addresses was obtained from Marketing System Group; the sample was drawn from an Address-Based Sampling frame created using the United States Postal Services Delivery Sequence File as its primary source. The sample frame included all known residential household addresses across the state with a 97% coverage rate. Known unoccupied housing units, vacation homes, and group living quarters were excluded from the sample. Drop-point addresses were expanded for inclusion in the sample frame.

On September 15, 2022, letters were mailed to all 5,000 addresses in the study sample. The initial invitation letter informed recipients about the survey and included instructions for completing the survey online. The initial mailing (included in the appendix) included links to the online instrument (URL and QR code) and a $5 bill as an incentive (Supplementary Figure S1). The letter specified that the household member over 18, who had the most recent birthday should complete the survey. The online survey was published using DCWorks' proprietary software and hosted on SESRC's secure web server array. One week after the initial mailing, a reminder postcard was sent to those who had not yet completed the survey).

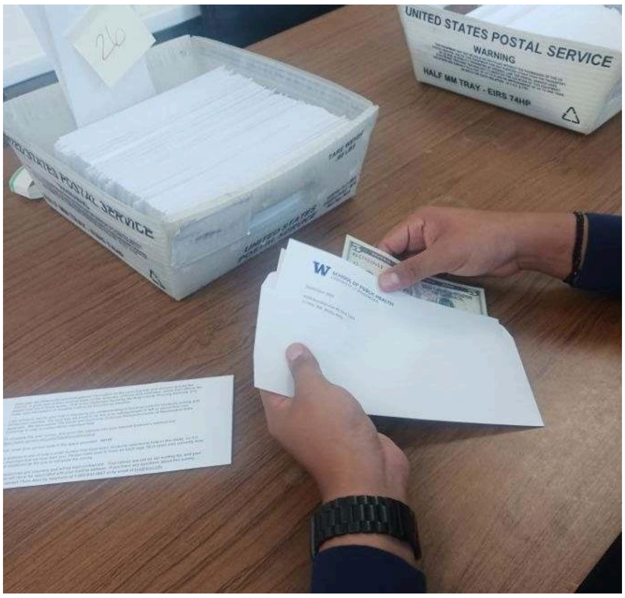

Supplementary Figure S1: The initial mailing to the sample of 5,000 included a $5 incentive.

On October 12, a paper questionnaire, cover letter and postage-paid return envelope were mailed to all addresses for which the survey had not yet been completed online. The paper survey was formatted as an 8-page color booklet and printed in SESRC's mail distribution center (Supplementary Figure S2).

A replacement questionnaire and another postage-paid return envelope was mailed to non-respondents approximately a month later, on November 14. A Spanish language translation of the letter was also included at that time. Paper questionnaires were coded by data collection staff and keyed twice into

ACI Open | DOI 10.1055/a-2695-8451 | © The Author(s).

the survey database to ensure accuracy. A final reminder letter in English and Spanish, as well as a link to the online survey was sent to non-respondents on December 1, 2022. Excess incentive funds from returned initial mailings were distributed as $1 bills to 900 households randomly selected from the 3422 households eligible for the final mailing. The survey closed on January 9th, 2023.

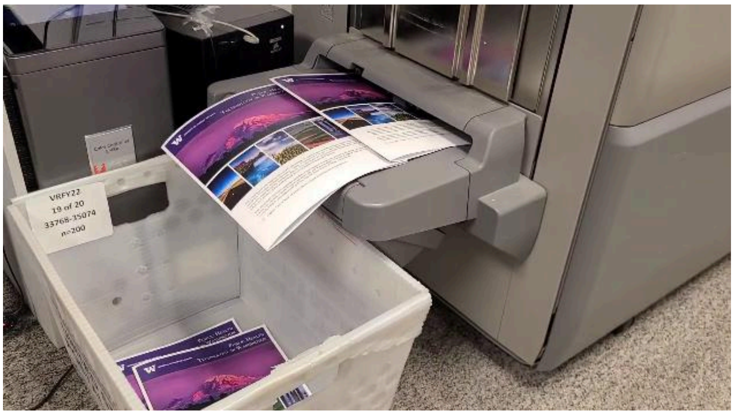

Supplementary Figure S2: Printing the survey as an 8-page booklet

The instructions specified that the household member over 18 years who had the most recent birthday should complete the survey. The initial mailing also included a $5 bill as an. Supplementary Table S1 shows the different mail distributions and the corresponding number of mailings sent. A copy of each mailing and the survey instrument are included in the supplemental file.
The survey instrument included single option, multiple selection and open-ended questions. A pilot survey was circulated to the WA DOH team, UW epidemiology students and other WA residents to gather feedback and suggestions. Feedback was incorporated, and changes were made to the content, wording, and flow of the survey.

Supplementary Table S1: Survey mail distribution timeline

Date	Description	Number mailed
September 15	Invitation Letter, Link + $5 Sent	5,000
September 22-23	Reminder Postcard, Link Sent	4,706
October 12-18	Paper Survey Sent	3,804
November 14-18	Paper Survey (copy) Sent	3,490
December 1-2	Final Reminder Sent (900 $1 bills randomly distributed)	3,422
Jan 9	Survey closed	--

Calibration Weights

The calibration weights were created such that the weighted proportions of individuals in the sample would simultaneously match both the marginal distribution of race/ethnicity and the joint distribution of age and sex in WA state. To improve stability, the race variable used in the creation of weights had only four categories (Hispanic of any race, non-Hispanic blacks, non-Hispanic whites and all other races). Weights were calculated using raking, implemented with the R survey package.[1,2]

ACI Open | DOI 10.1055/a-2695-8451 | © The Author(s).

Additional Results

Digital/Tech Readiness

The following survey items were compared with WA figures from the American Trends Panel Wave 88 Survey conducted by Pew Research Center in April 2021 (Pew Research Center, 2021a) (Pew Research Center, 2021b): confidence with digital devices, level of help needed when setting up new devices, smartphone ownership, and tech readiness. Overall, WA state digital readiness figures were close to the national proportions (Pew Research Center, 2021a). Supplementary Table S2 presents these descriptive data alongside the statewide figures.

Supplementary Table S2: Digital readiness characteristics of the survey sample compared to national data

<table>
<tr><th>Attribute</th><th>Values/Range</th><th>Sample # (%)</th><th>National Percentages*</th></tr>
<tr><td rowspan="5">Confidence with digital devices to do things online (Q01)</td><td>Very confident</td><td>915 (61.4%)</td><td>57%</td></tr>
<tr><td>Somewhat confident</td><td>410 (27.5%)</td><td>33%</td></tr>
<tr><td>Only a little confident</td><td>106 (7.1%)</td><td>7%</td></tr>
<tr><td>Not at all confident</td><td>49 (3.3%)</td><td>2%</td></tr>
<tr><td>Missing</td><td>11 (0.7%)</td><td>1%</td></tr>
<tr><td rowspan="5">Usually need help setting up new electronic device (Q02)**</td><td>Very true</td><td>155 (10.4%)</td><td rowspan="2">26%</td></tr>
<tr><td>True</td><td>223 (15.0%)</td></tr>
<tr><td>Slightly true</td><td>440 (29.5%)</td><td rowspan="2">73%</td></tr>
<tr><td>Not true at all</td><td>664 (44.5%)</td></tr>
<tr><td>Missing</td><td>9 (0.6%)</td><td>1%</td></tr>
<tr><td rowspan="3">Own a smartphone (Q03)</td><td>Yes</td><td>1409 (94.5%)</td><td>91%</td></tr>
<tr><td>No</td><td>71 (4.8%)</td><td>9%</td></tr>
<tr><td>Missing</td><td>11 (0.7%)</td><td>--</td></tr>
<tr><td rowspan="3">Tech readiness (Q01 and Q02)</td><td>Lower tech readiness</td><td>405 (27.2%)</td><td>30%</td></tr>
<tr><td>Higher tech readiness</td><td>1075 (72.1%)</td><td>69%</td></tr>
<tr><td>Missing</td><td>11 (0.7%)</td><td>--</td></tr>
</table>

* Pew Research Center American Trends Panel Wave 88 Survey, data collected April 12-18, 2021 (Pew Research Center, 2021a)
** Pew Research Center collected this question differently but comparably. In the WA data, “Very true” and “true” correspond to “Need someone else to set it up or show me how to use it” and “slightly true” and “not true at all” correspond to “am able to set it up and learn how to use it on my own” (Pew Research Center, 2021b)

To describe the overall digital readiness of respondents, we modified the Pew Research Center tech readiness measure (Pew Research Center, 2021b) as follows:

- “Lower tech readiness” was assigned to respondents who were either “not at all confident” or “only a little confident” using computers, smartphones, or other electronic devices to do things online, *or* usually needed help using new devices (i.e., answered "very true" or "true" to this question).
- “Higher tech readiness” was assigned to respondents who are “very confident” or “somewhat confident” using their digital devices to do the things they need to do online *and* are usually able to set up and learn how to use a new device on their own (i.e., answered “slightly true” or “not true at all” to this question).

ACI Open | DOI 10.1055/a-2695-8451 | © The Author(s).

Supplementary Figure S3 illustrates lower and higher tech readiness assignments of the WA survey sample. Over one-quarter (27.2%) of the WA survey sample was characterized as "lower tech readiness" and 72.1% were in the "higher tech readiness" group. For comparison, Pew characterized 30% of U.S. adults as having “lower tech readiness” and 69% as having “higher tech readiness”.

**Lower Tech Readiness**	**Higher Tech Readiness**
How confident are you using computers, smartphones, or other electronic devices to do things online?	How confident are you using computers, smartphones, or other electronic devices to do things online?
☑ Not at all confident **OR** ☑ Only a little confident	☑ Very confident **OR** ☑ Somewhat confident
**OR**	**AND**
Do you usually need help setting up new devices?	Do you usually need help setting up new devices?
☑ True **OR** ☑ Very true	☑ Slightly true **OR** ☑ Not true at all

Supplementary Figure S3: Definitions of Mutually Exclusive Lower and Higher Tech Readiness Groups

Who does not own a smartphone in WA State?

Seventy-one survey respondents (4.8%) indicated they do not own a smartphone; this is lower than the national average of 9% (Pew Research Center, 2021a). There were more individuals who did not own smartphones in the older age groups; 80+ and 70-79-year-olds reported not owning a smartphone at rates of 28.6% and 9.0%, respectively. Lower education levels and Eastern WA residents also had higher proportions of non-smartphone owners than would be expected if smartphone ownership was independent from demographics (4.8%). Supplementary Figure S4 illustrates these demographic differences.

ACI Open | DOI 10.1055/a-2695-8451 | © The Author(s).

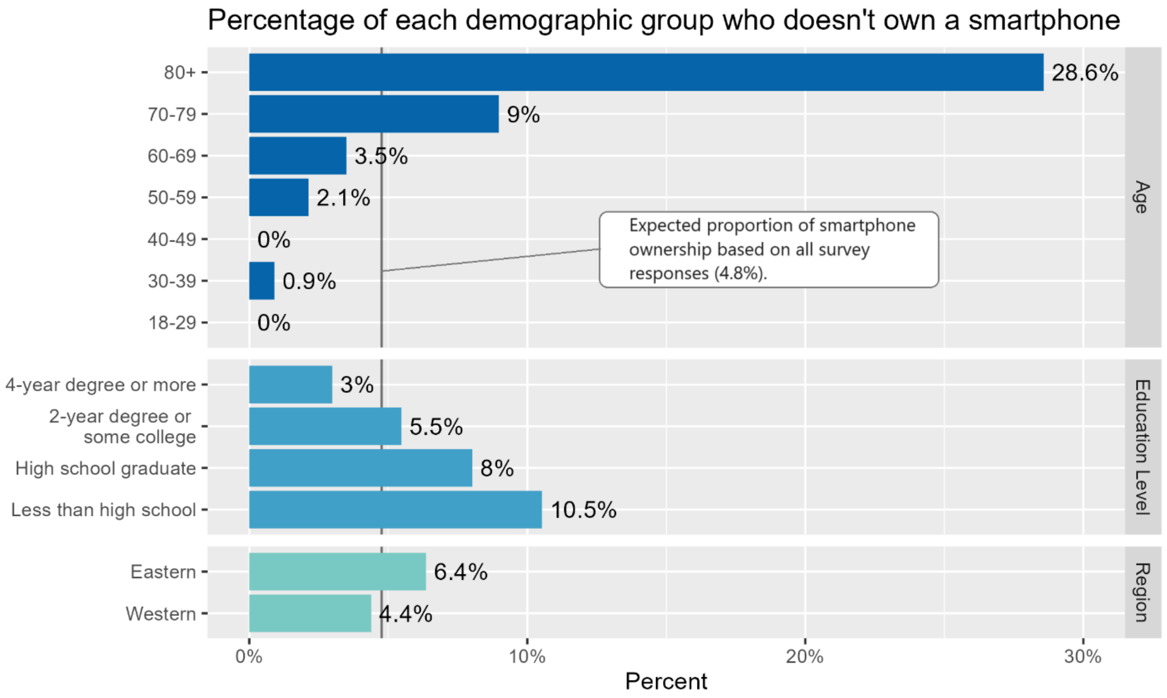

Supplementary Figure S4: Percentage of each demographic group who does not own a smartphone

Internet Access and Using Electronic Health Tools

Regarding access to the Internet, 96.8% of respondents were connected to the Internet which is slightly higher than the 91.5% reported in the 2021 ACS (Census Bureau, 2022). Most respondents reported using a cellular data plan (90.1%) and/or cable and fiber optic networks (87.8%) to access the Internet. Supplementary Figure S5 describes survey Internet access types compared to the ACS 2021 5-year results.

ACI Open | DOI 10.1055/a-2695-8451 | © The Author(s).

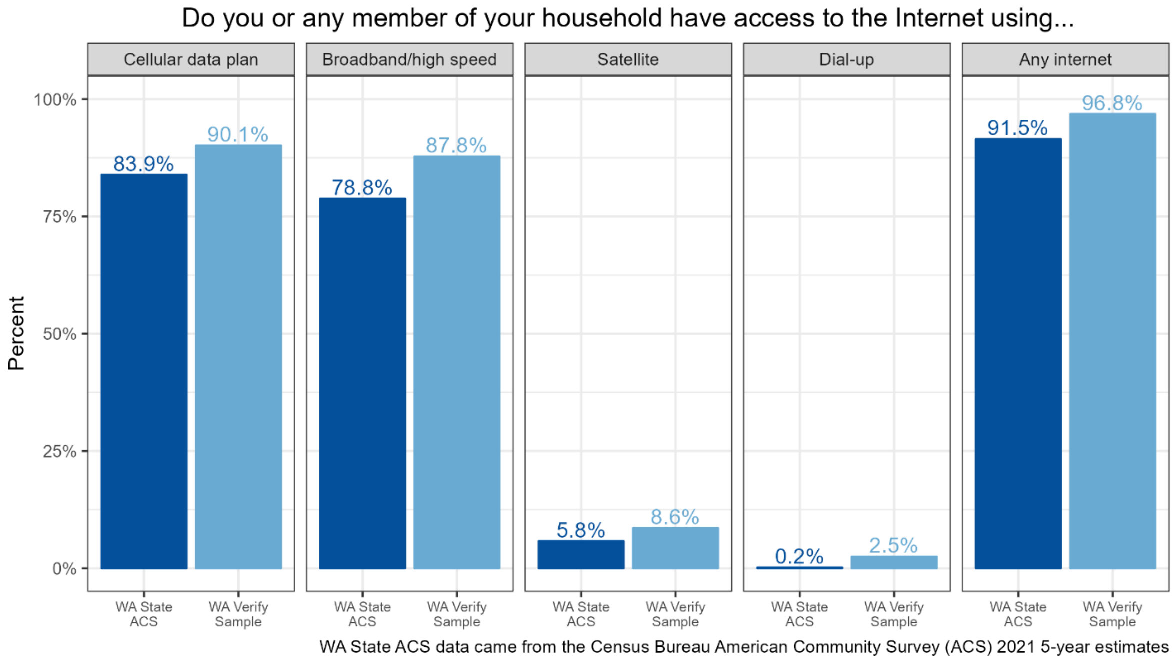

Supplementary Figure S5: Summary of internet access in survey sample and across the U.S. (Q04)

Regarding use of online tools, 88.3% of respondents reported using at least one online health tool, with use of online patient medical records or 'health portal' tools highest (83.6%), followed by tools for personal health tracking (e.g., heartrate monitors, step tracking, etc.) (55.0%). Supplementary Figure S6 summarizes survey respondents' use of electronic health tools.

ACI Open | DOI 10.1055/a-2695-8451 | © The Author(s).

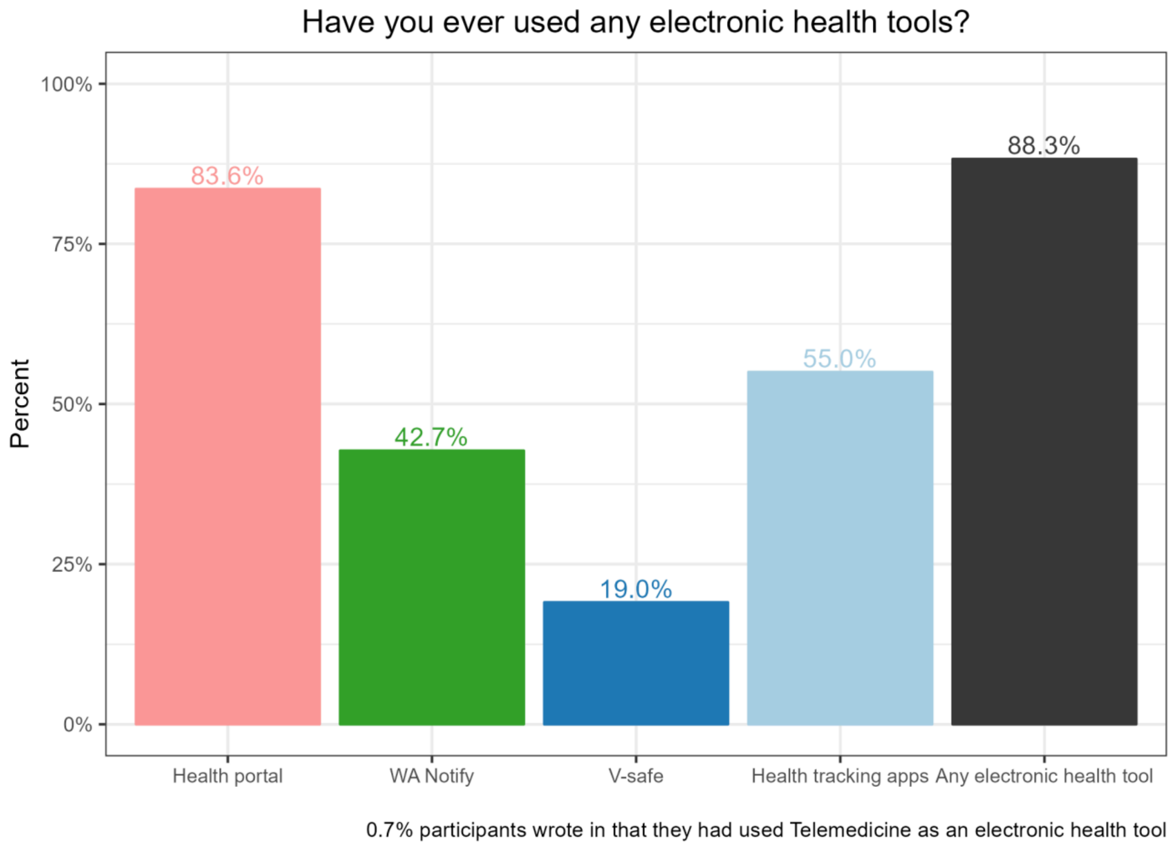

Supplementary Figure S6: Use of electronic health tools (Q05)

Predictors of WA Verify Awareness, Use & Adoption

To identify factors that may be associated with awareness of WA Verify, use of WA Verify, and willingness to adopt a tool like WA Verify weighted logistic regression was utilized. Weights account for both the sampling scheme and to better match the known population distribution of Washington state. Because the sample was a simple random sample of households, initial weights were proportional to the reported household size (and inversely proportional to the probability an individual in that household was sampled, conditional on the address being sampled). Post-stratification adjustments were applied to the weights such that the weighted sample distribution would match that of Washington state for both the joint age and sex distribution and the marginal distribution of race. Because some individuals didn't report demographic information, a hot-deck imputation strategy was utilized. Some respondents did not report age (N=64/1491), race (N=121/1491), or sex (N=115/1491). The variable with the least amount of missingness (age) was imputed first and subsequent variables were imputed based on the previously imputed values. Although 71 respondents did not report household size, to avoid small group sizes, this value was not used to impute other variables.

Each weighted logistic regression considered a single demographic or technology covariate and one of three outcome variables: 1) awareness of WA Verify (Supplementary Table S3), 2) reported use of WA Verify (Supplementary Table S4), and 3) willingness to use WA Verify (Supplementary Table S5). The

ACI Open | DOI 10.1055/a-2695-8451 | © The Author(s).

demographic covariates included age category, sex, combined race and ethnicity, and WA region (East/West,). The technology covariates were reported use of electronic health services (healthcare portals, personal health apps, etc.), access to the Internet, owning a smartphone, and level of tech-readiness.

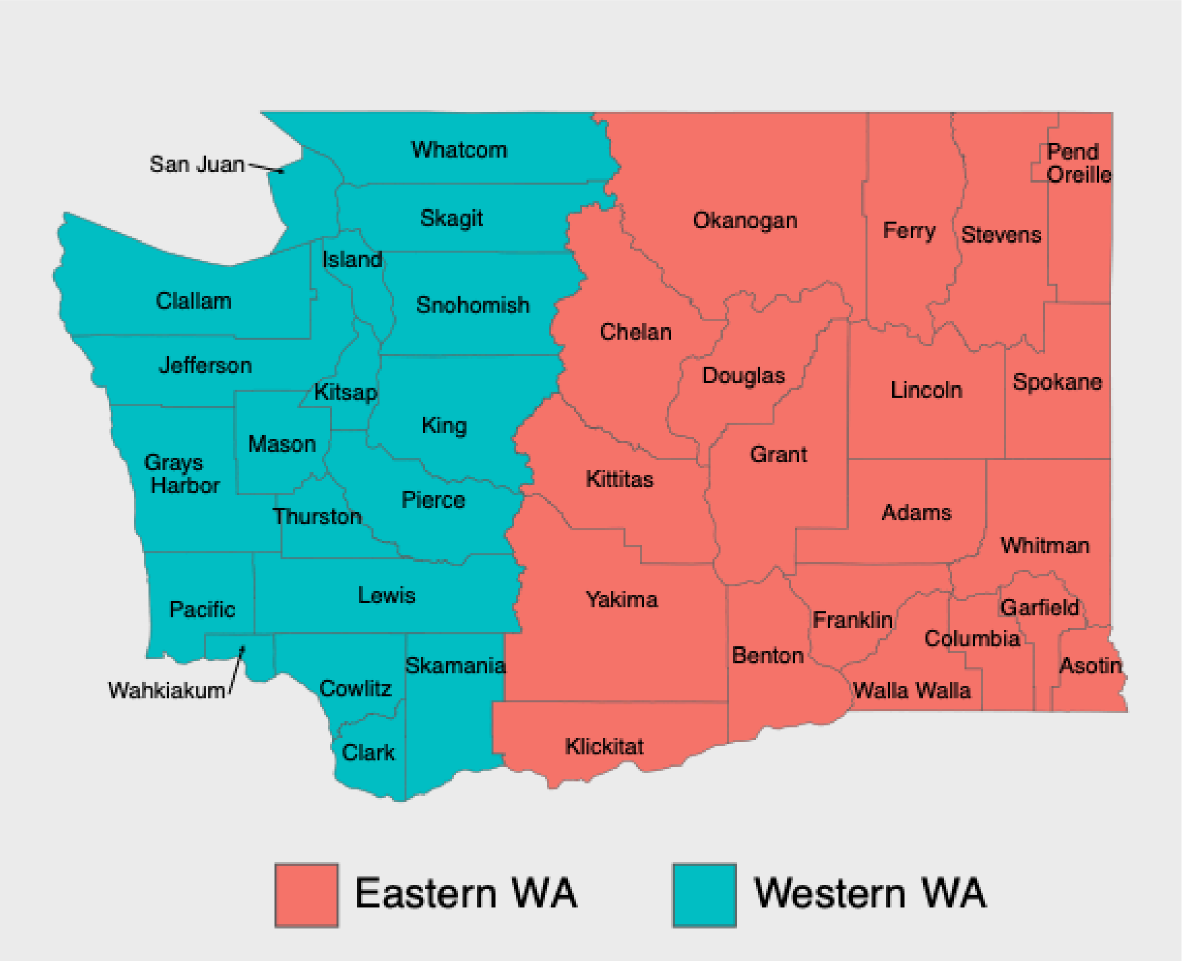

Supplementary Figure S7: Region of Washington by county.

Demographic Covariates and Predictors of Awareness, Use, and Willingness to Adopt WA Verify

With the exception of the 40-49 age group, older individuals tended to have lower odds of being aware of WA Verify, using WA Verify, and being willing to use WA Verify. Compared to those aged 18-29 years old, those who were 60-69 (OR = 0.53, 95%CI 0.30-0.92), 70-79 (OR = 0.40, 95%CI 0.22-0.71), or 80+ (OR = 0.24, 95%CI 0.12-0.48) had lower odds of reporting willingness to use WA Verify. However, compared to those aged 18-29 years old, those who were 40-49 years old had higher odds of reporting awareness of WA Verify (OR = 2.12, 95%CI 1.25-3.62) and use of WA Verify (OR = 2.00, 95%CI 1.06, 3.75).

Technology Covariates and Predictors of Awareness, Use, and Willingness to Adopt WA Verify

Relative to those who reported not using any electronic health services, those who did use these services were more likely to report awareness of WA Verify (OR =2.03, 95%CI 1.13-3.63), use of WA Verify (OR = 6.32, 95%CI 2.59-15.40), and willingness to use WA Verify (OR = 5.05, 95%CI 2.98-8.56). Relative to those who reported not owning a smartphone, smartphone owners were more likely to

ACI Open | DOI 10.1055/a-2695-8451 | © The Author(s).

report use of (OR = 9.26, 95%CI 2.18-39.34), willingness to use (OR =13.37, 95%CI 6.45-27.72), and awareness of WA Verify (OR = 3.50, 95%CI 1.71-7.17). Relative to those with higher tech readiness, those with lower tech readiness were less likely to be aware of (OR = 0.46, 95%CI 0.34-0.61), use (OR = 0.52, 95%CI 0.36-0.75), or be willing to use WA Verify (OR = 0.38, 95%CI 0.28-0.51).

Individuals who reported having access to internet were more likely to report willingness to use WA Verify (OR = 7.41, 95%CI 2.54-21.61) than those who did not have internet. Additionally, individuals living in Western WA were more likely than their eastern counterparts to report willingness to use WA Verify (OR = 2.06, 95%CI 1.45-2.93).

In summary, experience using electronic health services and smartphone ownership were significantly associated with awareness and use of WA Verify and willingness to adopt a tool like WA Verify. Although there were several significant findings, it should be noted that the confidence intervals for findings were quite wide and corrections for multiple testing were not made.

ACI Open | DOI 10.1055/a-2695-8451 | © The Author(s).

Supplementary Table S3: Odds Ratios for Awareness of WA Verify

Covariate	Baseline	Level	Weighted	Unweighted
Age Group	18-29	30-39	1.27, (0.75, 2.15)	1.34, (0.83, 2.17)
		40-49	**2.12, (1.25, 3.62)**	**1.95, (1.20, 3.19)**
		50-59	1.49, (0.88, 2.51)	1.40, (0.87, 2.25)
		60-69	1.20, (0.73, 1.97)	1.18, (0.75, 1.86)
		70-79	0.88, (0.52, 1.49)	0.79, (0.49, 1.28)
		80+	0.54, (0.28, 1.06)	**0.44, (0.23, 0.83)**
Has used electronic health services	No	Yes	**2.03, (1.13, 3.63)**	**3.17, (1.90, 5.27)**
Has internet access		Yes	2.79, (0.95, 8.26)	**4.01, (1.37, 11.72)**
Race	Black	Hispanic	1.05, (0.41, 2.71)	0.94, (0.40, 2.22)
		Other	1.28, (0.56, 2.94)	1.21, (0.56, 2.61)
		White	1.50, (0.69, 3.25)	1.16, (0.57, 2.38)
Region	East	West	1.34, (0.94, 1.92)	**1.48, (1.10, 2.01)**
Sex	Female	Male	0.83, (0.63, 1.09)	0.80, (0.63, 1.01)
Owns a smartphone	No	Yes	**3.50, (1.71, 7.17)**	**4.30, (2.24, 8.26)**
Tech readiness	Higher	Lower	**0.46, (0.34, 0.61)**	**0.41, (0.31, 0.54)**

Supplementary Table S4: Odds Ratios for WA Verify Use

Covariate	Baseline	Level	Weighted	Unweighted
Age Group	18-29	30-39	1.17, (0.63, 2.17)	1.62, (0.92, 2.86)
		40-49	**2.00, (1.06, 3.75)**	**2.16, (1.22, 3.80)**
		50-59	1.45, (0.78, 2.72)	1.66, (0.94, 2.93)
		60-69	1.06, (0.58, 1.94)	1.24, (0.72, 2.14)
		70-79	0.72, (0.37, 1.40)	0.86, (0.47, 1.59)
		80+	0.60, (0.25, 1.43)	0.66, (0.28, 1.55)
Has used electronic health services	No	Yes	**6.32, (2.59, 15.40)**	**7.15, (2.98, 17.13)**
Has internet access	No	Yes	2.27, (0.48, 10.69)	2.50, (0.49, 12.74)
Race	Black	Hispanic	1.28, (0.40, 4.11)	0.85, (0.30, 2.46)
		Other	1.41, (0.53, 3.80)	1.43, (0.57, 3.59)
		White	1.29, (0.51, 3.31)	1.33, (0.56, 3.17)
Region	East	West	1.44, (0.91, 2.28)	**1.72, (1.17, 2.53)**
Sex	Female	Male	0.97, (0.71, 1.33)	0.92, (0.70, 1.20)
Owns a smartphone	No	Yes	**9.26, (2.18, 39.34)**	**9.57, (1.97, 46.39)**
Tech readiness	Higher	Lower	**0.52, (0.36, 0.75)**	**0.48, (0.34, 0.67)**

Supplementary Table S5: Odds Ratios for Willingness to Use WA Verify

Covariate	Baseline	Level	Weighted	Unweighted
Age Group	18-29	30-39	0.62, (0.33, 1.14)	0.65, (0.37, 1.16)
		40-49	0.97, (0.52, 1.82)	0.90, (0.50, 1.64)

ACI Open | DOI 10.1055/a-2695-8451 | © The Author(s).

Covariate	Baseline	Level	Weighted	Unweighted
		50-59	0.65, (0.36, 1.18)	0.66, (0.38, 1.16)
		60-69	**0.53, (0.30, 0.92)**	**0.52, (0.31, 0.89)**
		70-79	**0.40, (0.22, 0.71)**	**0.39, (0.22, 0.67)**
		80+	**0.24, (0.12, 0.48)**	**0.26, (0.13, 0.50)**
Has used electronic health services	No	Yes	**5.05, (2.98, 8.56)**	**5.80, (3.75, 8.97)**
Has internet access		Yes	**7.41, (2.54, 21.61)**	**7.99, (2.73, 23.36)**
		Hispanic	0.95, (0.33, 2.78)	0.98, (0.35, 2.75)
Race	Black	Other	0.74, (0.28, 1.95)	0.82, (0.33, 2.09)
		White	0.65, (0.27, 1.61)	0.73, (0.31, 1.75)
Region	East	West	**2.06, (1.45, 2.93)**	**2.18, (1.60, 2.98)**
Sex	Female	Male	0.98, (0.73, 1.31)	0.97, (0.75, 1.26)
Owns a smartphone	No	Yes	**13.37, (6.45, 27.72)**	**12.32, (6.38, 23.79)**
Tech readiness	Higher	Lower	**0.38, (0.28, 0.51)**	**0.39, (0.29, 0.51)**

Additional regional results:

Feelings on Policies and regarding proof of vaccination and on portable electronic COVID-19 vaccine records

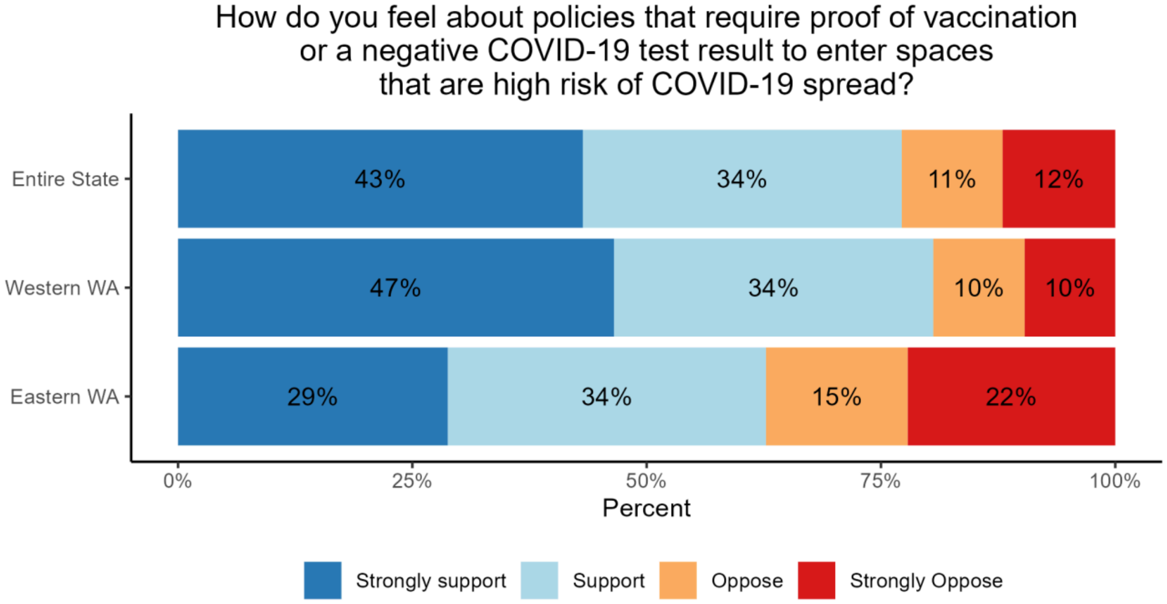

Supplementary Figure S8: Opinions regarding COVID-19 vaccination and testing policies by region. Reported percentages are for responses to "How do you feel about policies that require proof of vaccination or a negative COVID-19 test result to enter spaces that are high risk of COVID-19 spread?"

ACI Open | DOI 10.1055/a-2695-8451 | © The Author(s).

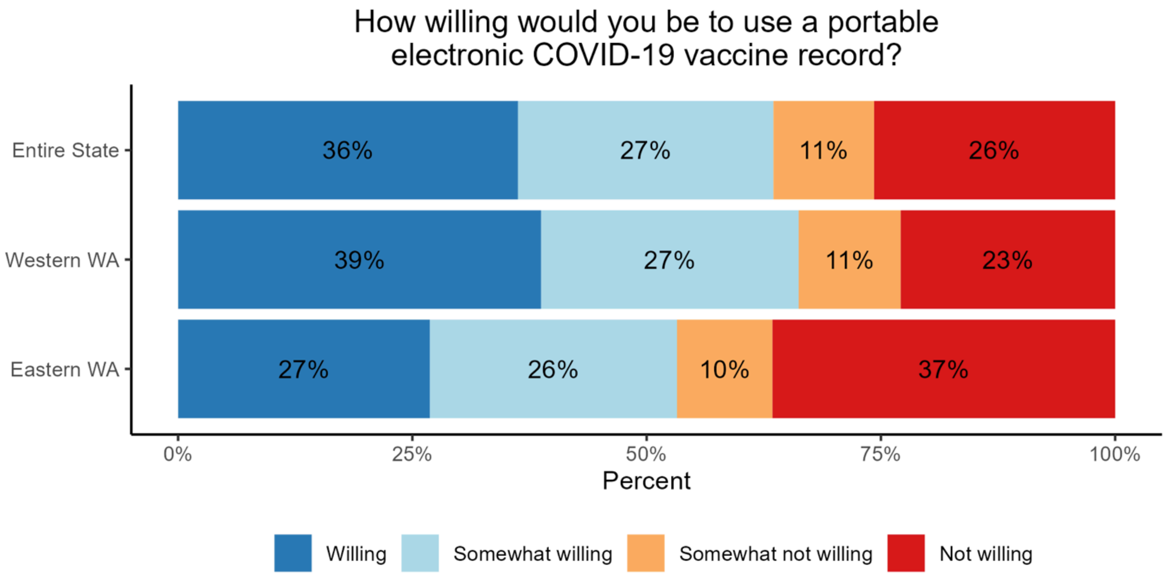

Supplementary Figure S9: Willingness to use an electronic vaccine record by WA region. Reported percentages are for responses to "How willing would you be to use a portable electronic COVID-19 vaccine record?". These percentages only include individuals who reported "No" to having used WA Verify or a similar tool.

References

AAPOR. (2023). *Standard Definitions (RR2 Calculation).* Retrieved June 01, 2023, from American Association of Public Opinions Research: https://aapor.org/wp-content/uploads/2023/05/Standards-Definitions-10th-edition.pdf

Census Bureau. (2022, 12 8). Retrieved 9 4, 2023, from American Community Survey 2017-2021 5-Year Data Release: https://www.census.gov/newsroom/press-kits/2022/acs-5-year.html

Pew Research Center. (2021a, April). *American Trends Panel Wave 88 (data collected April 12-18, 2021)*. Retrieved 2023, from Pew Research Center: https://www.pewresearch.org/internet/dataset/american-trends-panel-wave-88/

Pew Research Center. (2021b). *The Internet and the Pandemic.* Retrieved from https://www.pewresearch.org/internet/wp-content/uploads/sites/9/2021/09/PI_2021.09.01_COVID-19-and-Tech_FINAL.pdf

U.S. Census Bureau. (2020). *HISPANIC OR LATINO, AND NOT HISPANIC OR LATINO BY RACE*. Retrieved October 4, 2023, from Decennial Census, DEC Redistricting Data (PL94-171), Table P2: https://data.census.gov/table/DECENNIALPL2020.P2?q=P2:+HISPANIC+OR+LATINO,+AND+NOT+HISPANIC+OR+LATINO+BY+RACE

U.S. Census Bureau. (2021). *SEX BY AGE.* Retrieved October 4, 2023, from American Community Survey, ACS 5-Year Estimates Detaioled Tables, Table B01001: https://data.census.gov/table/ACSDT5Y2021.B01001?q=B01001:+Sex+by+Age

US CENSUS BUREAU. (2016). Characteristics of People by Language Spoken at Home [data table for 2016]. *American FactFinder*. Retrieved 09 15, 2023, from https://factfinder.census.gov/faces/tableservi

USPS. (2017). Retrieved from CDS User Guide: https://postalpro.usps.com/cds/User_Guide

ACI Open | DOI 10.1055/a-2695-8451 | © The Author(s).

WA Department of Health. (2023, 07 01). *Department of Health Data and Statisical Reports*. Retrieved from COVID-19 Vaccination Data: https://doh.wa.gov/data-statistical-reports/health-behaviors/immunization/covid-19-vaccination-data

ACI Open | DOI 10.1055/a-2695-8451 | © The Author(s).
